# The photosynthetic response of tobacco plants overexpressing ice plant aquaporin McMIPB to a soil water deficit and high vapor pressure deficit

**DOI:** 10.1007/s10265-013-0548-4

**Published:** 2013-01-31

**Authors:** Miki Kawase, Yuko T. Hanba, Maki Katsuhara

**Affiliations:** 1 Graduate School of Science and Technology, Kyoto Institute of Technology, Matsugasaki, Sakyo-ku, Kyoto, 606-8585 Japan; 2 Institute of Plant Science and Resources, Okayama University, Chuo, Kurashiki, Okayama 710-0046 Japan

**Keywords:** CO_2_ transporter, Atmospheric humidity, Mesophyll conductance, Mesophyll anatomy

## Abstract

We investigated the photosynthetic capacity and plant growth of tobacco plants overexpressing ice plant (*Mesembryanthemum crystallinum* L.) aquaporin McMIPB under (1) a well-watered growth condition, (2) a well-watered and temporal higher vapor pressure deficit (VPD) condition, and (3) a soil water deficit growth condition to investigate the effect of McMIPB on photosynthetic responses under moderate soil and atmospheric humidity and water deficit conditions. Transgenic plants showed a significantly higher photosynthesis rate (by 48 %), higher mesophyll conductance (by 52 %), and enhanced growth under the well-watered growth condition than those of control plants. Decreases in the photosynthesis rate and stomatal conductance from ambient to higher VPD were slightly higher in transgenic plants than those in control plants. When plants were grown under the soil water deficit condition, decreases in the photosynthesis rate and stomatal conductance were less significant in transgenic plants than those in control plants. McMIPB is likely to work as a CO_2_ transporter, as well as control the regulation of stomata to water deficits.

## Introduction

To perform leaf photosynthesis, CO_2_ must diffuse from the atmosphere to the site of carboxylation in stroma through leaf mesophyll. The diffusional conductance of CO_2_ through the stomata and mesophyll is called stomatal conductance (*g*_s_) and mesophyll conductance (*g*_m_), respectively, where the limitation of photosynthesis by *g*_m_ is almost equal, or even larger, than that by *g*_s_ (Yamori et al. 2006). In determining *g*_m_, conductance in the liquid phase, which is across the cell wall, plasma membrane, cytosol, chloroplast envelope, and stroma, is important. Among the possible factors affecting liquid phase conductance, the surface area of chloroplasts facing the intercellular air space, S_c_, is important as the active area for CO_2_ diffusion to chloroplast stroma (Hanba et al. 2004; Terashima et al. 2006, 2011).

Recent studies suggest that some plant aquaporins (water channel proteins that transport water molecules) in the plasma membrane and chloroplast envelope are CO_2_ transport candidates that reduce the diffusional resistance of CO_2_. To date, three aquaporins including NtAQP1, AtPIP1;2, and HvPIP2;1 have been shown to reduce the diffusional resistance of CO_2_ in leaves. Uehlein et al. (2003, 2008) showed that tobacco (*Nicotiana tabacum* L.) aquaporin NtAQP1 functions as a CO_2_ transporter by in vitro analysis using a heterologous expressing system in *Xenopus* oocytes, and also by in vivo analysis using RNA interference mediated decreases in NtAQP1. Recently, *Arabidopsis thaliana* aquaporin AtPIP1;2 was shown to be a CO_2_ transporter with the yeast heterologous expression system (Heckwolf et al. 2011). Hanba et al. (2004) and Flexas et al. (2006) reported the possibility of CO_2_ permeability on plant aquaporin in vivo; barley aquaporin HvPIP2;1 and tobacco aquaporin NtAQP1 overexpressing plants increased *g*_m_ more than control plants. However, tobacco aquaporin NtPIP2;1 was shown to have no CO_2_ permeability (Otto et al. 2010), suggesting that CO_2_ permeability differs largely between aquaporins. Further studies are needed to investigate the role of aquaporin on CO_2_ diffusion in leaves.

On the other hand, some aquaporins play a role in water transport in plants, which affects plant responses to drought. However, the relationship between aquaporin expression and plant responses to drought stress is controversial. Aharon et al. (2003) reported that tobacco plants overexpressing *Arabidopsis thaliana* aquaporin PIPb wilted faster than control plants under drought conditions. Barley aquaporin HvPIP2;1 overexpressing rice plants grew less under salt stress (Katsuhara et al. 2003). On the other hand, NtAQP1 anti-sense tobacco plants showed lower tolerance to water stress (Siefritza et al. 2002). Lowland rice overexpressing RWC3, which is strongly expressed in upland rice, achieved drought avoidance under drought stress (Lian et al. 2004).

Leaf photosynthetic responses to drought vary largely between species (Chaves et al. 2009), which is an important factor relating whole-plant responses to drought. Both a soil water deficit and atmospheric vapor water deficit (VPD) should be considered to understand plant photosynthetic responses to drought. Although the effect of a soil water deficit on the limitation of plant photosynthesis has been extensively studied (Chaves et al. 2009; Flexas et al. 2009), the effect of aquaporin on the photosynthetic response to a soil water deficit has scarcely been studied. Although the effects of VPD on the regulation of *g*_s_ have been extensively observed (Day 2000; Shirke and Pathre 2004; Warren 2008), the role of aquaporin on the regulation of *g*_s_ to changes in VPD has not been studied yet. Aquaporin overexpressing plants tend to increase *g*_s_ under non-water-stressed conditions (Flexas et al. 2006; Hanba et al. 2004), suggesting the role of aquaporin in stomatal regulation. Furthermore, specific aquaporins are highly expressed in guard cells (Fraysse et al. 2005; Kaldenhoff et al. 1995; Otto and Kaldenhoff 2000; Sun et al. 2007). The controversy among previous studies for the relationship between the level of aquaporin expression and plant responses to drought may be due to differences in the stomatal response to VPD and/or a soil water deficit via aquaporin, which was scarcely considered in previous studies. In this study, we investigated the role of aquaporin on the plant response to a water deficit by changing VPD and the soil water content to clarify the effect of aquaporin on stomatal regulation to a water deficit.

In this study, we focused on the role of the ice plant (*Mesembryanthemum crystallinum* L.) aquaporin, McMIPB (Accession L36097), in leaf photosynthesis. *M*. *crystallinum* is native to southern and eastern Africa, and is a halophyte with a developmentally programmed switch from C_3_ photosynthesis to crassulacean acid metabolism (CAM) that is accelerated by salinity and drought (Adams et al. 1998). McMIPB has been identified as a PIP1 type aquaporin (Yamada et al. 1997) and is mainly located at xylem parenchyma in the ice plant (Kirch et al. 2000). Yamada et al. (1995) described that ice plant aquaporin *McMIPB* transcript products were unchanged relative to the other ice plant aquaporin transcripts, *McMIPA* and *McMIPC*, during salt stress treatment. We estimate that McMIPB plays an important role under drought conditions in maintaining water balance in the plant body and its photosynthetic capacity.

The purpose of the present study was to investigate the role of McMIPB, a new candidate for enhancing CO_2_ diffusion and regulating stomata, on leaf photosynthesis, plant growth, and plant responses to a water deficit. We compared control plants and overexpression McMIPB plants grown under three different conditions: (1) a well-watered growth condition, (2) a well-watered and temporal higher VPD condition, and (3) a soil water deficit growth condition. The localization of McMIPB inside leaves, photosynthetic parameters, and plant growth were compared between controls and overexpressed plants grown in the well-watered condition to determine the roles of McMIPB in CO_2_ diffusion, leaf photosynthesis, and growth. Leaf mesophyll anatomy was analyzed to investigate the effects of mesophyll anatomy on CO_2_ diffusion. The responses of leaf photosynthetic parameters, levels of McMIPB protein levels, and plant growth were then investigated for temporal higher VPD and the water deficit growth condition to clarify the roles of McMIPB in stomatal regulation to a water deficit.

## Materials and methods

### Transformation of tobacco

A full length of *McMIPB* (Accession No. L36097) cDNA was inserted downstream from a 35S-promoter in the expression vector pBI121. The transformation of the tobacco leaf disk with *Agrobacterium* methods, regeneration, and the selection of transgenic plants with kanamycin (100 mg/L) were performed as previously described (Horsch et al. 1985). T_2_ generation of McMIPB overexpressing tobacco, line 8884, was made based on non-transgenic tobacco plants, line SR. The parents of line 8884 were homozygotes.

### Plant growth

Seeds of tobacco plants were sowed on an agar medium and grown in a growth chamber (LPH-350S, NK system, Japan) under the following conditions: temperatures of 25/18 °C (day/night), a photoperiod of 16/8 h (day/night), relative humidity of 70 %, and PPFD of 300 μmol m^−2^ s^−1^. When cotyledons expanded, plants were transplanted to 0.5 L plastic pots filled with culture soil (green soil, Tankyo, Japan) and akadama pumice (7:3, volume ratio). They were watered daily, and were fertilized once a week with a Hoagland solution. We used small plants with 5–6 leaves grown for 1 month after seeding until experiments were started. Tobacco plants had shade leaves in the present study under the growth conditions of the growth chamber.

### Generation of an antibody and measurement of the protein levels of aquaporin

A polyclonal antibody was raised against a synthetic oligopeptide corresponding to QPSQYEM in loop C of *McMIPB*. Leaf disks (1.77 cm^2^ × 2) were obtained from the central part of the lamina to analyze the protein levels of aquaporin. Total protein extraction, SDS-PAGE, and protein blotting were performed as described previously (Katsuhara et al. 2002). Bands detected in the Western blot were scanned and analyzed using software (Densitograph, ATTO, Tokyo, Japan). The antibody recognized bands in SR lines as well as 8884 lines. The total level of bands was mentioned as McMIPB.

### Identification of the localization of aquaporin by immunostaining

Some sections of 2 × 3 mm were obtained from leaves, and were fixed into a solution containing 3.7 % formaldehyde, 5.0 % acetic acid, and 50 % ethanol. Sections were blocked with 1 % bovine serum albumin in PBS–Tween-20 (0.2 %) and were then reacted with a solution containing 1/500 of McMIPB antibodies. Sections were reacted with a 1/500 solution of the second antibody anti-rabbit IgG (Alexa Fluor^®^ 488 Conjugate, Life Invitrogen, Japan). Localization of the aquaporin detected by the McMIPB antibody (McMIPB) was observed using a fluorescence microscope (BX51, Olympus, Japan). To analyze the occurrence of non-specific responses, negative control analysis was examined. Immunostaining was performed on leaf sections as described previously, without the step of incubation with the first antibodies.

### Gas exchange measurement and estimation of mesophyll CO_2_ conductance

Gas exchange measurements were performed for intact leaves under PPFD of 700 μmol m^−2^ s^−1^ and ambient CO_2_ concentration of 360 ppm for all experiments. Measurements were started 2 h after the chamber’s lights were on. For experiment 1, gas exchange was measured at ambient VPD (551 ± 107 Pa) for SR and 8884 lines grown under the well-watered condition. Plants were irrigated every day. The leaf temperature was 25 °C. For experiment 2, gas exchange was compared between ambient VPD (890 ± 130 Pa) and higher VPD (1630 ± 120 Pa) for SR and 8884 lines grown under the well-watered growth condition. Plants were irrigated every day. Leaf temperatures were 25 and 28 °C for ambient and higher VPD measurements, respectively. For experiment 3, plants were grown under the well-watered condition (watered every day) for 1 month after seeding, and then when 5–6 leaves expanded, the drought treatment (watered once a week) was performed for 1 month. Changes in gas exchange parameters by the drought treatment were compared between SR and 8884 lines. Gas exchange measurements were performed every day for 7 days from the next day of the last irrigation of the drought treatment. On the 6th day, plants were re-watered after gas exchange measurements were completed for the day. Leaf temperature was 26 °C, and VPD was 1002 ± 85.5 Pa.

Gas exchange parameters were calculated according to the modified method of von Caemmerer and Evans (1991). Mesophyll conductance (*g*_m_) was estimated for intact leaves by concurrent measurement of gas exchange and the carbon isotope ratio (Hanba et al. 2002) using the laboratory-constructed system described by Hanba et al. (1999). The isotope method is considered the most reliable for the estimation of *g*_m_. In the present study, *g*_m_ was calculated using the simplified equation reported by Scartazza et al. (1998):

1 $$ g_{m} = \frac{{(b - a_{i} )\frac{A}{{C_{a} }}}}{{(\Updelta_{i} - \Updelta ) - \frac{{f\Upgamma^{*} }}{{C_{a} }}}} $$

where Δ is the observed carbon isotope discrimination, Δ_i_ is the expected carbon isotope discrimination assuming infinite *g*_m_, *A* the assimilation rate, *C*_a_ the CO_2_ partial pressure in ambient air, *a*_i_ the carbon isotope discrimination during CO_2_ diffusion/hydration into water (1.8 %), and *b* is the carbon isotope discrimination caused by carboxylation by Rubisco and PEP carboxylase (28.2 ‰). The symbols *f* and $\Upgamma^{*}$ represent discrimination with photorespiration and a CO_2_ compensation point without day respiration. Recently, *g*_m_ has been estimated in detail with changes in light intensity, CO_2_ concentration, leaf anatomy, activity of photosynthetic enzymes, and for different genotypes, where the effect of *f* on *g*_m_ is considered (Barbour et al. 2010; Kodama et al. 2011; Tazoe et al. 2009, 2011). Here, we assumed the effect of *f* was negligible because we examined it with the same light intensity, CO_2_ concentration, and plant materials. Measurements were repeated twice for each leaf sample. Δ was calculated from carbon isotope ratios of CO_2_ in the air leaving and entering the gas exchange chamber. CO_2_ samples were collected using dry ice–ethanol and liquid nitrogen traps. The carbon isotope ratio was determined with an isotope mass spectrometer (Finnigan MAT 252, Bremen, Germany). Analysis of the carbon isotope ratio was performed as described by Hanba et al. (1999).

### Analysis of leaf mesophyll anatomy

For experiment 1, leaf mesophyll anatomy was determined using light micrographs. Some sections of 2 × 3 mm leaves were fixed in 2.5 % glutaraldehyde and 2 % osmium tetroxide, and were then embedded in a replacement of Spurr’s resin (Low Viscosity Resin kit, TAAB, United Kingdom). Transverse sections 700 nm thick were stained with a 1 % toluidine blue solution. Light micrographs were taken using a digital camera (VB-7000, Keyence, Japan), which was attached to a light microscope (BX51, Olympus, Japan). Anatomical characteristics were determined from digitized images of micrographs taken at 200× magnification. Surface areas of mesophyll cells and chloroplasts exposed to intercellular air spaces per unit leaf area (*S*_mes_ and *S*_c_) were estimated for transverse sections as described previously (Hanba et al. 2002).

### Measurements of plant growth and water content

For experiment 1, stem diameters and dry weights of shoots were measured in 4-month-old plants. Leaf area was measured in 2-month-old plants. For experiment 2, relative water content (RWC) was measured in 2-month-old plants. For experiment 3, stem diameters, dry weights of shoots and roots, and plant heights were measured in 14-week-old plants.

Stem diameter was measured using a digital caliper (CD-20C, Mitutoyo, Japan) 10 cm from the soil surface for experiment 1, and 5 cm from the soil surface for experiment 3. To determine the dry weight, shoots or roots were dried at 70 °C for 48 h and weighed. Leaf area was calculated by scanning leaves used for gas exchange measurements, using Image J software (National Institutes of Health, MD, USA). To obtain RWC, leaf disks (1.77 cm^2^) were harvested and their fresh weight (FW) was determined. After being soaked over 12 h in ion-exchanged water at 4 °C, their turgid weight (TW) was measured. They were then dried at 70 °C for 24 h and their dry weight (DW) was obtained. RWC was then calculated as follows:

$$ {\text{RWC}} = \left[ {({\text{FW}} - {\text{DW}})/({\text{TW}} - {\text{DW}})} \right] \times 100\,\% . $$

### Measurements of Rubisco content

Leaf disks (1.77 cm^2^) were obtained from the central part of the lamina, and were homogenized immediately at 4 °C in HEPES buffer (pH 7.5) containing 0.2 % Triton X-100, 0.7 % 2-mercaptoethanol, 2 mM monoiodoacetic acid, 25 % glycerol, 6 % lithium lauryl sulfate, 1 % polyvinyl polypyrrolidone, and 1 mM phenylmethylsulfonylfluoride. The homogenate was immediately centrifuged, and the supernatant was boiled for 5 min in Laemmli (1970) buffer. Proteins in supernatants were separated by SDS-PAGE, and gels (12.5 %) were stained with a solution (Coomassie brilliant blue, Bio-Rad Laboratories, Japan). The level of Rubisco subunits was determined spectrophotometrically by scanning the gel with a scanner (GT7600U, Epson, Tokyo, Japan). Results were analyzed using software (Densitograph, ATTO, Tokyo, Japan). The level of Rubisco was determined using BSA (BSA standard set, Bio-Rad, Japan) as the standard. The Rubisco content may be different from estimations using the extraction method by Makino et al. (1985).

### Statistical analysis

Differences in mean values between transgenic and control plants were analyzed using an unpaired *t* test and paired *t* test.

## Results

### The level and localization of McMIPB

**Fig. 1:**
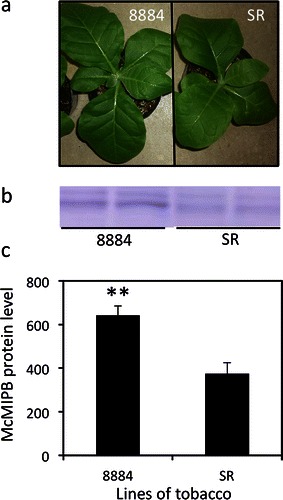
Images of whole plant and protein levels of aquaporins detected by the McMIPB antibody (McMIPB). **a** Images of lines 8884 and SR grown for 1 month after seeding, **b** levels of McMIPB, and **c** intensity of McMIPB protein levels that were densitometrically quantified by Western analysis, where values are mean ± SE from different plants (*n* = 3–5). Statistical analysis was done using an unpaired *t* test. *Asterisks above bars* indicate that the means of transgenic plants are significantly different from the SR line (*p* < 0.01)

The size of the tobacco plant was larger in line 8884 than that in line SR (Fig. 1a). The protein band detected by the McMIPB antibody (McMIPB) was observed in both SR and 8884 lines (Fig. 1b). The level of McMIPB was 71 % higher in 8884 than that of SR (Fig. 1c).

**Fig. 2:**
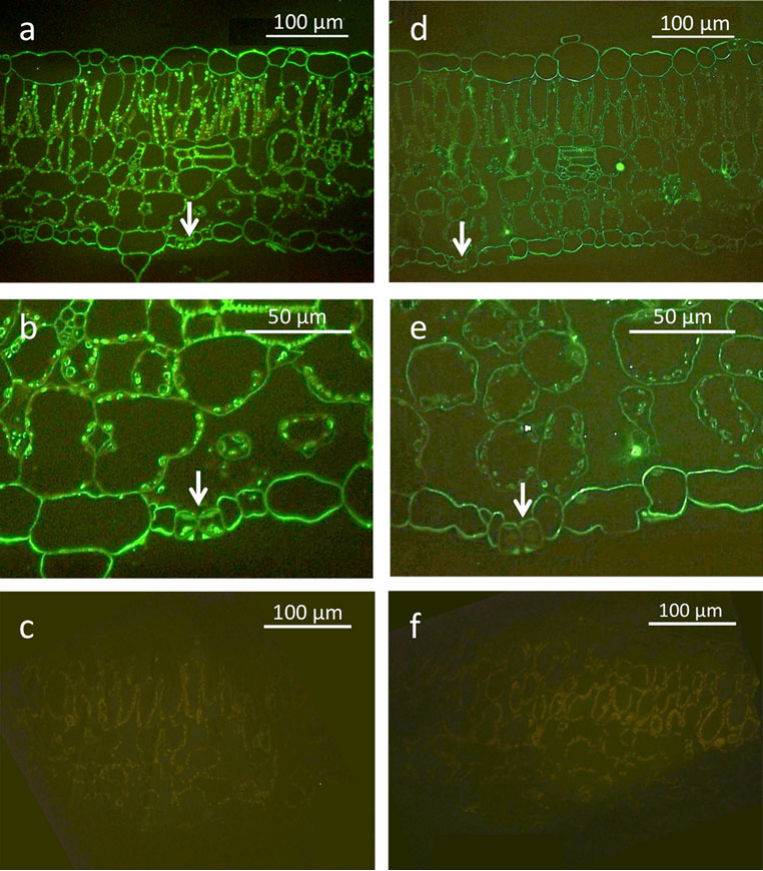
Localization of McMIPB in the leaf mesophyll of tobacco plants grown under the well-watered condition. **a**–**c** Line 8884, and **d**–**f** line SR. Florescence micrographs were taken at ×400 magnification. **c**, **f** Negative controls of lines 8884 and SR. *Arrows* show stomata

In line 8884, the strongest McMIPB signals were detected at chloroplasts in mesophyll cells and also at chloroplasts in stomata (Fig. 2a, b). Signals were also detected at vascular bundles and epidermal cells. In the SR line, weak signals were detected at chloroplasts in mesophyll cells (Fig. 2d, e). Signals at chloroplasts in stomata were very weak. Significant signals were detected at vascular bundles and epidermal cells. Mesophyll cells were slightly tinged with red due to the auto-fluorescence of chloroplasts in both lines, but non-specific responses were not detected in either line (Fig. 2c, f).

### Characteristics of transgenic plants grown under the well-watered condition

**Fig. 3:**
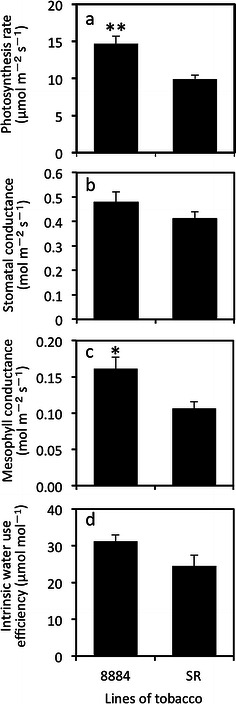
Gas exchange parameters in tobacco plants grown under the well-watered condition measured at ambient VPD. **a** Photosynthesis rate, **b** stomatal conductance, **c** mesophyll conductance, and **d** intrinsic water use efficiency, which is the ratio of the photosynthesis rate to stomatal conductance (*A*/*g*_s_). Values are mean ± SE of the two replicates from 4 to 5 different plants (*n* = 8–10). Statistical analysis was done using an unpaired *t* test. *Asterisks above bars* indicate that the means of transgenic plants are significantly different from the SR line (*p < 0.05, **p < 0.01)

In comparison with the SR line, stem diameter of the 8884 line increased by 16 % (Table 1). The dry weight of the shoots in line 8884 was 62 % higher than that in the SR line. Although not significant, leaf area tended to be larger in line 8884. Transgenic plants showed a significantly higher photosynthesis rate (by 48 %), higher mesophyll conductance (by 52 %), and higher stomatal conductance (by 16 %) than those of the SR line (Fig. 3a–c). Intrinsic water use efficiency, which is calculated from the ratio of the photosynthesis rate to stomatal conductance (*A*/*g*_s_), was 27 % higher in line 8884 than that in line SR (Fig. 3d).

**Table 1:** Characteristics of tobacco plants grown under the well-watered condition

Characteristics	8884	SR
Leaf thickness (μm)	313 ± 9	280 ± 36
Mesophyll porosity (%)	48 ± 2	49 ± 3
*S*_mes_ (m^2^ m^−2^)	14.5 ± 1.3	13.2 ± 1.1
*S*_c_ (m^2^ m^−2^)	8.8 ± 0.6	8.9 ± 0.4
Stem diameter (mm)	8.12 ± 0.27*	7.02 ± 0.03
Dry weight of shoot (g)	11.4 ± 1.1*	7.0 ± 0.9
Leaf area (cm^−2^)	13.1 ± 1.1	11.7 ± 6.0
RWC (%)	64.7 ± 0.9	68.2 ± 3.2
Rubisco content (g m^−2^)	1.57 ± 0.10	1.78 ± 0.20

Values are mean ± SE from four different plants (*n* = 4). Statistical analysis was done using an unpaired *t* test

* Means of transgenic plants are significantly different from the SR line (*p* < 0.05)

### Photosynthesis under ambient and higher VPDs

**Fig. 4:**
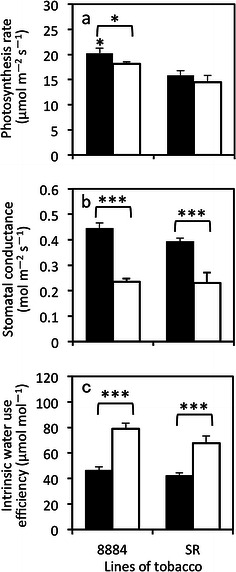
Gas exchange parameters in tobacco plants grown under the well-watered condition, measured at ambient and higher VPD. **a** Photosynthesis rate, **b** stomatal conductance, **c** intrinsic water use efficiency, which is the ratio of the photosynthesis rate to stomatal conductance (*A*/*g*_s_). Values are mean ± SE of the two replicates from 3 to 5 different plants (*n* = 6–10). *Filled bars* and *open bars* are ambient VPD and higher VPD, respectively. Statistical analysis was done using an unpaired *t* test to determine the significance between lines and a paired *t* test between treatments. *Asterisks above bars* indicate that the means of transgenic plants are significantly different from the SR line in the same treatment, and *asterisks above the arcs over the bars* indicate significant differences between ambient and higher VPD in the same line (*p < 0.05, ***p < 0.001)

The photosynthesis rate of line 8884 was smaller at the higher VPD than the ambient VPD, but it was similar between ambient and higher VPDs for line SR (Fig. 4a). The photosynthesis rate was 10 % lower at the higher VPD in line 8884. On the other hand, stomatal conductance was significantly smaller at the higher VPD than ambient VPD in lines 8884 and SR, where the decrease was more in line 8884 (by 47 %) than that in line SR (by 41 %, Fig. 4b). Intrinsic water use efficiency was higher at the higher VPD, where the increase was greater in line 8884 (by 71 %) than that in line SR (by 60 %, Fig. 4c).

Under the same VPD, the photosynthesis rate of line 8884 was significantly higher (by 28 %) than that of line SR at ambient VPD. The photosynthesis rate of line 8884 was slightly higher than that of line SR at the higher VPD (by 25 %). The stomatal conductance of line 8884 was slightly higher than that of line SR at ambient VPD, although it was almost the same as that of line SR at the higher VPD. Intrinsic water use efficiency was almost the same value at ambient VPD. However, in the higher VPD condition, line 8884 had a slightly higher value (by 17 %) than that of line SR.

### Rubisco content and mesophyll anatomy

**Fig. 5:**
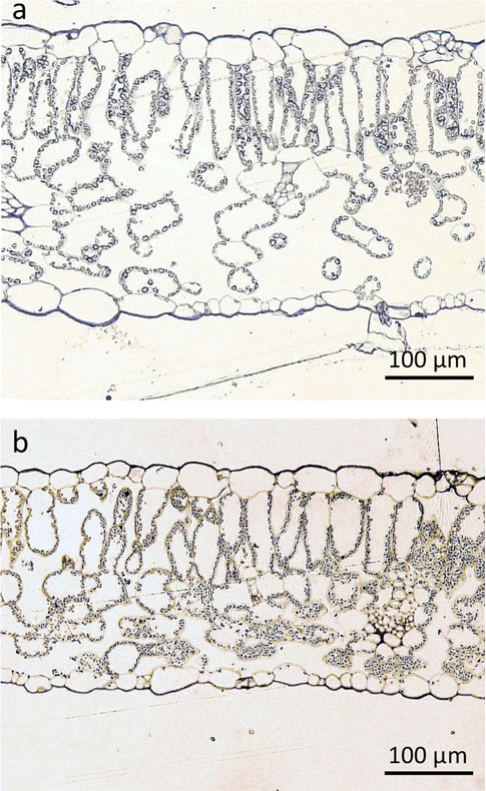
Light micrographs of leaf sections of tobacco plants, taken at ×200 magnification. **a** Line 8884, **b** line SR

Rubisco content was similar between lines SR and 8884 grown under the well-watered condition (Table 1). Leaf thickness, mesophyll porosity, *S*_mes_, and *S*_c_ were similar between SR and 8884 lines (Table 1; Fig. 5). In both SR and 8884 lines, stomata were observed at epidermal cells on both the adaxial and abaxial sides of leaves (Fig. 5). Leaves have one layer of palisade tissue. Mesophyll cells were relatively loosely packed. Most of the surface of mesophyll cells facing intercellular air spaces was covered with chloroplasts.

### Photosynthesis response to the soil water deficit

**Fig. 6:**
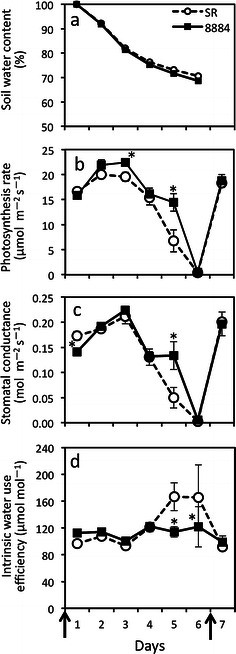
Changes in gas exchange parameters in tobacco plants under progressing soil water deficits and at re-watering. Plants were exposed to a water deficit for 1 month (irrigated once a week). Gas exchange measurements were performed from 1 to 5 days after the last irrigation date (*left arrow*) and at the re-watering date (*right arrow*). **a** Soil water content was estimated relative to pot weight at the first day from the last irrigation, **b** photosynthesis rate, **c** stomatal conductance, and **d** intrinsic water use efficiency, which is the ratio of the photosynthesis rate to stomatal conductance (*A*/*g*_s_). Values are mean ± SE from five different plants (*n* = 5). Statistical analysis was done using an unpaired *t* test. *Asterisks above bars* indicate that the means of transgenic plants are significantly different from the SR line on the same day (*p* < 0.05)

Soil water content gradually decreased, reaching about 70 % at 6 days after the last irrigation for both SR and 8884 lines (Fig. 6a). The pattern of changes in the photosynthesis rate with time was similar to that of stomatal conductance for both SR and 8884 lines (Fig. 6b, c). The photosynthesis rate and stomatal conductance tended to be higher in line 8884 than those in line SR from 2 to 6 days after the last irrigation. At 5 days after the last irrigation, both the photosynthetic rate and stomatal conductance were significantly higher in line 8884 than those in line SR (113 and 169 % higher than that of SR, respectively). Line 8884 showed lower intrinsic water use efficiency than line SR 5 and 6 days after the last irrigation (Fig. 6d). On the 7th day, the photosynthesis rate and stomatal conductance recovered due to re-watering. There was no difference in the degree of recovery between lines 8884 and SR.

**Fig. 7:**
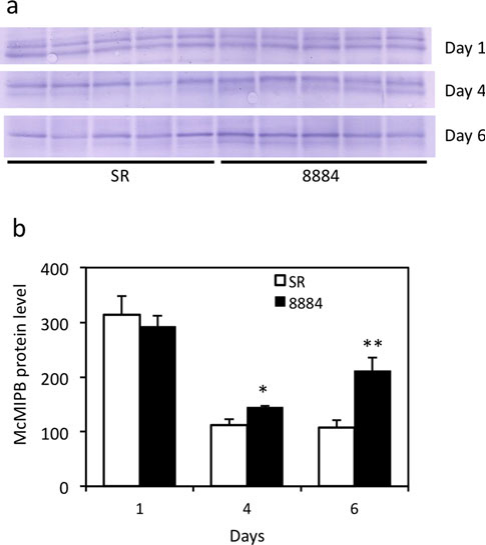
McMIPB protein levels of tobacco leaves grown under progressing soil water deficits. **a** McMIPB protein level and **b** intensity of McMIPB protein levels that were densitometrically quantified by Western analysis, where values are mean ± SE from five different plants (*n* = 5). Statistical analysis was done using an unpaired *t* test. *Asterisks above bars* indicate that the means of transgenic plants are significantly different from the SR line on the same day (*p < 0.05, **p < 0.01)

McMIPB protein levels changed with the soil water deficit treatment (Fig. 7a, b). As water stress became severe, McMIPB protein levels became higher in line 8884 than those in line SR. Plant height and stem diameter were similar between lines 8884 and SR when grown under the soil water deficit condition (Table 2). The dry weight of shoots under the soil water deficit condition was slightly higher in line 8884, while there was no change in the dry weight of roots (Table 2).

**Table 2:** Plant growth of tobacco plants grown under the water-stressed condition

Plant growth	8884	SR
Plant height (cm)	14.2 ± 2.2	14.6 ± 2.6
Stem diameter (mm)	4.66 ± 0.22	4.65 ± 0.11
Dry weight of shoot (g)	3.16 ± 0.12	3.04 ± 0.13
Dry weight of root (g)	0.62 ± 0.03	0.62 ± 0.02

Values are mean ± SE from five different plants (*n* = 5). Statistical analysis was done using an unpaired *t* test. The means of transgenic plants were not significantly different from the SR line (*p* > 0.05)

## Discussion

### Increased aquaporin levels enhanced photosynthesis and growth under the well-watered condition

Starting with the report of Terashima and Ono (2002), the number of studies concerning the CO_2_ permeability of aquaporin is now increasing (reviews by Flexas et al. 2012; Kaldenhoff et al. 2008; Katsuhara et al. 2008; Maurel 2007). The leaf photosynthesis rate in Eucalyptus trees was enhanced in transgenic plants overexpressing radish aquaporin RsPIP2;1 (Tsuchihira et al. 2010), supporting the role of aquaporin in leaf photosynthesis. In this study, McMIPB overexpressing tobacco plants grown under the well-watered condition increased mesophyll conductance (Fig. 3c), which suggest the CO_2_ permeability of aquaporin localized in mesophyll cells and chloroplasts (Fig. 2a, d). McMIPB levels and photosynthetic capacity were closely related (Figs. 1, 3). The increase in the photosynthesis rate in McMIPB overexpressing plants (Fig. 3a) was not due to changes in mesophyll anatomy and levels of carboxylation enzymes, because the Rubisco content and *S*_c_ were similar between McMIPB overexpressing and control plants (Table 1). Therefore, we suggest that McMIPB in mesophyll cells and chloroplasts should enhance CO_2_ diffusion into chloroplasts of mesophyll cells and thus increase the photosynthetic rate. The localization of aquaporin in plasma membranes of mesophyll cells and chloroplasts was shown for tobacco aquaporin NtAQP1, which had CO_2_ permeability and enhanced CO_2_ diffusion and photosynthesis rate (Uehlein et al. 2008).

CO_2_ permeability differs among aquaporins. Tobacco aquaporin NtAQP1 enhanced CO_2_ diffusion, whereas NtPIP2;1 did not (Otto et al. 2010). CO_2_ permeability may not depend on the sub-type of aquaporin (PIP1 or PIP2, Katsuhara, unpublished data), while the co-expression of PIP2 with PIP1 may enhance the channel activity of PIP1. The different combinations of PIP1 and PIP2 in the subunits of aquaporin tetramers may affect the CO_2_ permeability of aquaporin (Flexas et al. 2012). McMIPB is classified as PIP1, with possible partners of PIP2 aquaporin McMIPC or McMIPE in the subunits of tetramers.

Stem diameter and dry weight in McMIPB overexpressing tobacco was significantly higher than those of control plants (Table 1), possibly due to increases in the leaf photosynthetic capacity. Plant heights were shown to drastically increase in tobacco plants overexpressing *A. thaliana* aquaporin PIP1b (Aharon et al. 2003), although NtAQP1 overexpressing tobacco plants did not show significant changes in growth over control plants (Flexas et al. 2006). Differences between studies may be due to exogenous or endogenous aquaporin, or may be partly affected by the plant size. In the present study, we used small plants with a small number of leaves with large leaf areas, where shoot growth was largely determined by leaf growth. Increases in leaf photosynthesis may have directly enhanced the growth of the whole plant through enhancements in leaf growth in the present study.

### Increased aquaporin levels affect the stomatal response to a water deficit

Both transgenic and control plants exposed to higher VPD showed a drastic decline in *g*_s_ (Fig. 4b), which is in line with previous reports that demonstrated declines in *g*_s_ at higher VPD for non-transgenic plants (Day 2000; Shirke and Pathre 2004; Warren 2008). We should note that an increase in leaf temperature by 3 °C at the higher VPD may have partly affected the stomatal response to higher VPD. Declines in *g*_s_ from ambient to higher VPD were more significant in overexpressing McMIPB plants. Declines in the transpiration rate from ambient to higher VPD were also larger in overexpressing McMIPB plants (data not shown). These results suggest that the sensitivity of *g*_s_ to temporal atmospheric water deficits may be higher in overexpressing McMIPB plants than that of control plants, which may moderate water loss from the leaves and increase water use efficiency (Fig. 3d).

On the other hand, when exposed to a soil water deficit, declines in the photosynthesis rate and stomatal conductance with time were lower in overexpressing McMIPB plants than those in control plants, at 5 days in particular (Fig. 6b, c). Higher McMIPB levels were obtained at 4 days in overexpressing McMIPB plants than those in control plants (Fig. 7b). These results suggest that a high aquaporin level relates to stomatal regulation to a soil water deficit, which induces less sensitivity of stomata to a soil water deficit, resulting in lower water use efficiency in overexpressing McMIPB plants (Fig. 6d). Stomatal aperture may be affected by aquaporin level via changes in turgor pressure of guard cells. The transpiration rate was much higher in overexpressing McMIPB plants at 5 days (data not shown), which is in line with results by Siefritza et al. (2002) in which the transpiration rate of control plants was larger than NtAQP1-antisense tobacco plants under drought conditions.

The results of a temporal atmospheric water deficit and prolonged soil water deficit suggest that the effect of aquaporin on stomatal sensitivity is different between atmospheric and soil water deficit. Warren (2008) showed that *g*_s_ was decreased by both atmospheric and soil water deficits, while *g*_m_ was decreased by a soil water deficit only. We obtained similar *g*_m_ between higher and ambient VPD for both overexpressed and control plants (data not shown), which supports the above results by Warren (2008). The lower sensitivity of *g*_s_ to a soil water deficit in overexpressing McMIPB plants may compensate partly for the decrease in CO_2_ supply by decreasing *g*_m_. Although the mechanisms are unclear, there may be a co-relationship between *g*_s_ and *g*_m_ through aquaporin, which may regulate the balance of CO_2_ supply to chloroplasts and water loss from leaves.

Some aquaporins are highly expressed in guard cells (Fraysse et al. 2005; Kaldenhoff et al. 1995; Otto and Kaldenhoff 2000; Sun et al. 2007). Tobacco aquaporin NtAQP1 was shown to localize in chloroplasts of guard cells, and this NtAQP1 has CO_2_ permeability (Uehlein et al. 2008). In this study, the McMIPB signal strongly appeared in the chloroplasts of guard cells in overexpressing McMIPB plants (Fig. 2), where McMIPB aquaporin should have CO_2_ permeability. These aquaporins may affect stomatal regulation, as well as decrease the resistance of photosynthetic pathways due to their CO_2_ permeability in guard cells.

The dry weight of shoots exposed to a soil water deficit was slightly higher in overexpressing McMIPB plants, while there was no change in the dry weight of roots (Table 2). These results support the report of Katsuhara et al. (2003), which shows a higher shoot/root ratio in overexpressing HvPIP2;1 rice plants under salt stress. The McMIPB aquaporin in the present study may play a role in water transport; the high hydraulic conductivity raised by aquaporin in the roots may support water transport with a low root mass in transgenic plants (Katsuhara et al. 2003).

### Relationship between mesophyll anatomy and aquaporin expression

Hanba et al. (2004) demonstrated that barley aquaporin HvPIP2;1 overexpressing rice plants showed anatomical changes (*S*_mes_, *S*_c_, mesophyll porosity, stomatal density, stomatal size). Tsuchihira et al. (2010) reported changes in the leaf morphology of Eucalyptus trees overexpressing RsPIP2;1. However, Flexas et al. (2006) reported that tobacco endogenous aquaporin NtAQP1 overexpressing tobacco plants showed no anatomical changes, and argued that anatomical changes may be due to the transformation by an exogenous aquaporin. In Table 1 and Fig. 5, overexpressing McMIPB tobacco plants showed no significant change in leaf thickness, mesophyll porosity, *S*_mes_, or *S*_c_. We suggest that anatomical changes in aquaporin overexpressing plants may be indirect ones, which may be the result of plant acclimation to some physiological changes such as water relations in transgenic plants. If aquaporin causes water stress in plants, leaf anatomy would change accordingly.

## Conclusions

The higher photosynthesis rate (by 48 %) and mesophyll conductance (by 52 %) in transgenic tobacco plants overexpressing ice plant aquaporin McMIPB than those in control plants under the well-watered growth condition suggests the CO_2_ permeability of McMIPB localized in mesophyll cells and chloroplasts. McMIPB should be the fourth aquaporin candidate for enhancing CO_2_ diffusion in leaves. Plants overexpressing aquaporin have low sensitivities to photosynthesis and *g*_s_ to a prolonged soil water deficit, but have slightly high sensitivities to photosynthesis and *g*_s_ to a temporal higher VPD, which is a new finding. These results suggest that McMIPB may be effective in compensating for the decrease in CO_2_ supply via stomatal regulation under a soil water deficit, but not so under a high VPD. Extensive studies will be needed to understand the role of aquaporin in photosynthesis, particularly in CO_2_ and water diffusion via mesophyll and stomatal regulation.

## Abbreviations

*g*_s_: Stomatal CO_2_ conductance
*g*_m_: Mesophyll CO_2_ conductance
McMIP: Aquaporin, which is an ice plant (*Mesembryanthemum crystallinum* L.) membrane intrinsic protein
McMIPB: Aquaporin detected by the McMIPB antibody
*S*_mes_: Surface area of mesophyll cells facing intercellular airspaces
*S*_c_: Surface area of chloroplasts facing intercellular airspaces
PIP: Aquaporin, which is a plasma membrane intrinsic protein
RWC: Relative water content
VPD: Vapor pressure deficit
